# Interplay Between Microglia and Alzheimer’s Disease—Focus on the Most Relevant Risks: APOE Genotype, Sex and Age

**DOI:** 10.3389/fnagi.2021.631827

**Published:** 2021-04-08

**Authors:** Yanting Chen, Tingting Hong, Feng Chen, Yuanhong Sun, Yan Wang, Lili Cui

**Affiliations:** ^1^Guangdong Key Laboratory of Age-Related Cardiac and Cerebral Diseases, Department of Neurology, Affiliated Hospital of Guangdong Medical University, Zhanjiang, China; ^2^Department of Pharmacology and Neuroscience, University of North Texas Health Science Center, Fort Worth, TX, United States

**Keywords:** microglia, Alzheimer’s disease, sex, aging, tau, amyloid β, apolipoprotein E

## Abstract

As the main immune cells of the central nervous system (CNS), microglia regulates normal development, homeostasis and general brain physiology. These functions put microglia at the forefront of CNS repair and recovery. Uncontrolled activation of microglia is related to the course of neurodegenerative diseases such as Alzheimer’s disease. It is clear that the classic pathologies of amyloid β (Aβ) and Tau are usually accompanied by the activation of microglia, and the activation of microglia also serves as an early event in the pathogenesis of AD. Therefore, during the occurrence and development of AD, the key susceptibility factors for AD—apolipoprotein E (APOE) genotype, sex and age—may further interact with microglia to exacerbate neurodegeneration. In this review, we discuss the role of microglia in the progression of AD related to the three risk factors for AD: APOE genotype, sex and aging. APOE-expressing microglia accumulates around Aβ plaques, and the presence of APOE4 may disrupt the phagocytosis of Aβ aggregates and aggravate neurodegeneration in Tau disease models. In addition, females have a high incidence of AD, and normal female microglia and estrogen have protective effects under normal conditions. However, under the influence of AD, female microglia seem to lose their protective effect and instead accelerate the course of AD. Aging, another major risk factor, may increase the sensitivity of microglia, leading to the exacerbation of microglial dysfunction in elderly AD. Obviously, in the role of microglia in AD, the three main risk factors of APOE, sex, and aging are not independent and have synergistic effects that contribute to the risk of AD. Moreover, new microglia can replace dysfunctional microglia after microglial depletion, which is a new promising strategy for AD treatment.

## Introduction

As the resident macrophages of the central nervous system (CNS; Gomez Perdiguero et al., 2015), microglia play an essential role in brain homeostasis, neuroinflammation and neurodegenerative diseases (Salter and Stevens, 2017). As the main neuroimmune sentinels in the brain (Daneman, 2012), microglia maintain the dynamic balance of the internal environment by continuously sensing changes in the external environment (Nimmerjahn et al., 2005). These immune detection and defense functions put microglia at the forefront of CNS repair and recovery. When the normal course of neurological development is disrupted, many neurological diseases can occur. Uncontrolled microglial activation is associated with the development of neurodegenerative diseases (Hansen et al., 2018).

As an age-related neurodegenerative disease, Alzheimer’s disease (AD) is the most common cause of dementia (Weuve et al., 2014). It is related to progressive cognitive decline and memory loss (Weuve et al., 2014). AD affects approximately 10% of the 65-year-old population and 50% of the 85-year-old population (Hebert et al., 2003). In the next few decades, this number is expected to increase significantly (Weuve et al., 2014). AD is characterized by the extracellular deposition of amyloid plaques, consisting predominantly of amyloid β (Aβ) peptides, and the intraneuronal accumulation of neurofibrillary tangles comprising aggregated, hyperphosphorylated tau protein (Serrano-Pozo et al., 2011). In addition to Aβ plaques and tau neurofibrillary tangles, neuroinflammation is also considered to be a key feature of AD pathology (Shi and Holtzman, 2018). As the main source of proinflammatory cytokines, microglia are the key mediators of neuroinflammation (Colonna and Butovsky, 2017). Single microglia sequencing confirmed the existence of a high proportion of activated microglia in APP transgenic mice (Sierksma et al., 2020). In AD-related lesions, the glial hyperplasia response may lead to the loss of cells after plaque and neurofibrillary tangle-accumulation (Ke et al., 2005). In contrast, chronic gliosis may also promote the accumulation of plaques and tangles before the onset of AD, leading to the progression of the disease (Herrup, 2010). These findings suggest that microglia may actively participate in the pathogenesis of AD. APOE genotype, sex and aging, the main risk factors for AD, affect the progression of AD and may interact with microglia to further aggravate neurodegeneration. Targeting microglia may be a preventive intervention to delay the progression of AD. In light of some new findings, in this review article, we will explore the role of microglia in the brain and how they interact with the factors of APOE genotype, sex, and aging to exacerbate AD.

## Physiological Function of Microglia

### Microglia in the Adult Brain

Microglia maintain and repair the damage to neural networks, which are the foundation of healthy brain development and function (Ginhoux et al., 2013). Unlike, monocytes derived from bone marrow, the maintenance and local expansion of microglia depend on self-renewal (Ajami et al., 2007; Ginhoux et al., 2010). Mature microglia are in a resting state and are characterized by multiple branches and processes (Nimmerjahn et al., 2005). Through two-photon imaging of the cerebral cortex *in vivo*, Nimmerjahn et al. (2005) found that microglia constantly observe the surrounding micro environment in their presumed resting state. It is worth noting that microglia are also sensors for brain pathology and can be quickly activated by minor pathological changes in the CNS (Kreutzberg, 1996). The phagocytic activity of activated microglia increases significantly to effectively remove neuronal debris (Stence et al., 2001).

### Microglia in the Developing Brain and the Difference Between Sexes

**Figure 1:**
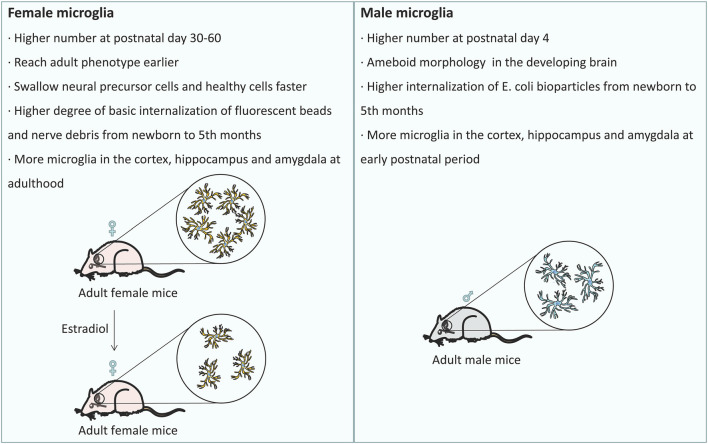
Sex differences in the development of microglia: the differences in the number of microglia and their distribution among the brain regions at different developmental stages are related to sex. Females exhibit a higher rate of phagocytosis of neural precursor cells and healthy cells than males, and females have more phagocytic microglia than males. After treatment with estradiol, the number of phagocytic microglia in female mice decreased to the typical level observed in male mice.

Microglia originate from red bone marrow progenitor cells in the yolk sac (Gomez Perdiguero et al., 2015). Microglial development occurs for a certain period of time and developing microglia undergo gradual changes to regulate the homeostasis of the brain (Matcovitch-Natan et al., 2016). In the developing brain, microglia repeatedly form short-term contacts with synapses, eliminating excess synaptic structures (Tremblay et al., 2010). Microglia not only participate in synapse pruning (Stevens et al., 2007; Paolicelli et al., 2011) and eliminate redundant neural precursor cells (Cunningham et al., 2013) but also promote neuronal apoptosis and eliminate dead cells (Marin-Teva et al., 2004; Wakselman et al., 2008). These functions give microglia, the ability to monitor neuronal activity and regulate synaptic plasticity. Studies in mice have shown that sex is linked to significant differences in microglia during and after development (Schwarz and Bilbo, 2012). For example, males have a significantly larger number of microglia in the early postnatal development period (postnatal day 4), while females have more activated microglia in the late developmental stages of adolescence and adulthood (postnatal day 30–60; Figure 1; Ruggiero et al., 2018). To our surprise, there are more microglia with amoeboid morphology in males, while female microglia tend to reach the adult phenotype earlier than male microglia (Figure 1; Lenz et al., 2013; Villapol et al., 2019). In addition, females have a higher rate of phagocytosis of neural precursor cells and healthy cells than males (Figure 1; Nelson et al., 2017). Microglia isolated from the brains of newborn female animals exhibited a higher degree of basic internalization of fluorescent beads and nerve debris than male microglia (Figure 1; Yanguas-Casas et al., 2020). In contrast, the microglia of male newborn animals exhibited higher internalization of *E. coli* bioparticles than the microglia of female newborn animals (Figure 1; Yanguas-Casas et al., 2020). However, these sex differences disappeared in microglia isolated from the brains of adults (5 months; Yanguas-Casas et al., 2020). Given the above findings, we believe that the sex differences in microglia are mainly reflected during the developmental stage. Compared with male mice, female mice have slight but significant differences in gene expression in different brain regions (Sala Frigerio et al., 2019). In the early postnatal period, male mice have more microglia in the cortex, hippocampus and amygdala than female mice (Figure 1; Schwarz et al., 2012). However, in adulthood, female mice have significantly more microglia in these brain regions than male mice (Figure 1; Schwarz et al., 2012). The gene expression of a large number of cytokines/chemokines and their receptors changes significantly with development and is highly dependent on sex (Schwarz et al., 2012). Based on the above results, we believe that the sex differences in microglia are mainly reflected during the developmental stage, and female microglia may have a protective effect during the development.

### Microglia in the Aging Brain

**Figure 2:**
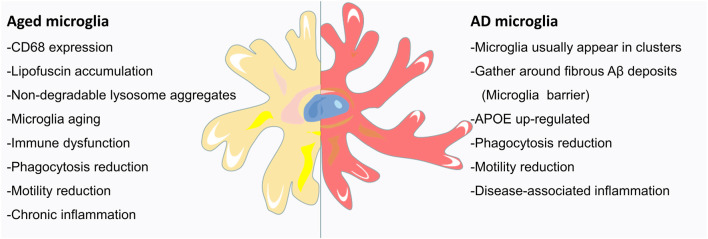
Microglia in the aging AD brain: the signs of aging are an increase in CD68 expression and the accumulation of lipofuscin. Aged mice have large microglia with short, thick dendrites, few branches, signs of malnutrition, and decreased phagocytotic and locomotor abilities. Age-related myelin fragmentation overloads the microglial lysosomal system, and the accumulation of lipids and nondegradable lysosomal aggregates leads to microglial senescence and immune dysfunction. As AD is a disease, closely related to aging, the microglia of the AD brain usually appear in clusters and gather around deposits of Aβ fibrils (discussed in the following section). With aging, the protruding movement of microglial processes is significantly reduced, and microglial coverage is reduced, leading to an increase in Aβ fibril hotspots. In addition to the upregulation of APOE, AD microglia also showed enhanced aging characteristics. Microglia respond to brain tissue damage that accumulates in AD and during aging, leading to increased inflammation, further reduction in phagocytic and motor abilities, and neuronal communication disorders.

During brain development, microglia guide neurons for proper CNS formation; in adulthood, microglia maintain tissue homeostasis; and in old age, microglia may become proinflammatory and finally exhausted (Figure 2; Valdés-Ferre et al., 2020). However, we currently know very little about microglia in the aging brain. Aging is one of the main risk factors for neurodegenerative diseases and is marked by an increase in CD68 expression and the accumulation of lipofuscin (Figure 2; Weber et al., 2015; Safaiyan et al., 2016). Zhao et al. (2020) found that the immunoreactivity of CD68 in the brains of 24-month-old mice was significantly higher than that in the brains of 3-month-old mice, confirming the effect of aging on the activation of microglia (Zhao et al., 2020). Microglial process speed significantly decreases with age (Streit et al., 2004; Olah et al., 2018). The changes in the phagocytic activity of senescent microglia *in vitro* were similar to those of microglia purified from aged brains (Yanguas-Casas et al., 2020). *In vivo* imaging of young, adult, and aging mice showed that in addition to a slight increase in cell density, microglial morphology and behavior also changed (e.g., larger cell bodies, shorter dendrites, thicker dendrites, fewer branches, and decreased phagocytic ability and motor ability; Figure 2; McGeer et al., 1987; Itagaki et al., 1989; Styren et al., 1990). During aging, the deposition of myelin in the parenchyma may exceed the processing capacity of microglia, leading to the accumulation of lipids and non-degradable lysosome aggregates (Figure 2). This observation has been confirmed: with age, myelin fragmentation increased, microglia entered a state of dysfunction, and insoluble lipofuscin-like lysosomal inclusion bodies appeared (Safaiyan et al., 2016). Age-related myelin fragments overload the microglial lysosome system, resulting in microglial aging and immune dysfunction (Figure 2; Safaiyan et al., 2016). Aging causes chronic inflammation of microglia (Figure 2; Hammond et al., 2019). With age, the function of microglia decreased, the normal brain monitoring function is gradually lost, and the pathological conditions of the brain may not be detected and processed in time. Therefore, the dysfunction of microglia in the aging brain may lead to age-related cognitive decline and neurodegenerative diseases.

### Microglial Phenotypes

Previous studies have divided microglia into resting (M0), proinflammatory (M1), and proresolution (M2) microglia by purifying isolated cells *in vitro*, but these phenotypes are highly dynamic (Ransohoff, 2016). When homeostasis in the nervous system is disrupted, microglia rapidly change their phenotype, which is often referred to as microglial activation (Ransohoff and Perry, 2009). Among these phenotypes, M1 microglia have cytotoxic effects on neurons and oligodendrocytes cultured *in vitro*, while M2 cells have phagocytic abilities and can promote neurite growth (Kigerl et al., 2009; Hu et al., 2012). Some studies have suggested that long-term activation of M1 and suppression of the M2 state are the basis of the inflammatory phenotype in AD and other chronic neurodegenerative diseases (Cherry et al., 2014). However, given the recent revelations regarding the complexity and dynamics of microglia, the M0/M1/M2-classifications based on *in vitro* studies may be too simple. In fact, recent single-cell transcriptome studies have revealed several different microglial subsets and cellular states in aging and disease, including “disease-associated microglia” (DAM; Keren-Shaul et al., 2017) “microglial neurodegenerative disease” (MGnD; Krasemann et al., 2017) and “activation response microglia (ARM)” (Sala Frigerio et al., 2019). Among these subsets, DAM may be a protective phagocytic microglial cell population (Keren-Shaul et al., 2017), while MGnD is a dysfunctional microglial phenotype (Krasemann et al., 2017). Analyses of whole-tissue RNA-seq and single-cell nuclear RNA-seq datasets show that the lack of a DAM response in human microglia occurs in AD tissues, but not in other neurodegenerative diseases (Srinivasan et al., 2020). To understand whether a microglial phenotypic switch from M0 (homeostatic) to MGnD (neurodegenerative) is related to neuritic dystrophy in AD, Krasemann et al. (2017) used P2ry12 and Clec7a monoclonal antibodies to distinguish M0 and MGnD microglia in brains from APP-PS1 mice and humans with AD (Krasemann et al., 2017). As a result, they found Clec7a^+^P2ry12^—^microglia associated with Aβ plaques in APP-PS1 mice (Krasemann et al., 2017). The phenotype of Clec7a^+^ microglia was similar to that of MGnD microglia, found in APP-PS1 models and during aging (Krasemann et al., 2017). Although the role of microglia in the pathogenesis of AD is a field of intensive research, whether the DAM and MGnD phenotypes are the same and their effects on the CNS (harmful or beneficial) may depend on the process and stage of the disease.

## Microglia and The Main Risk Factors for AD

In the developing brain, microglia can destroy invading microorganisms and remove harmful cellular debris (Kreutzberg, 1996). In general, microglia usually return to a resting state after the pathogen is cleared. Nevertheless, if the CNS is exposed to activation stimulation for a long time, microglia will enter a dysfunctional state (Kreutzberg, 1996). Microglia change significantly in brain pathology, undergoing a complex multistage activation process (Kettenmann et al., 2011) and changing from highly branched resting cells to reactive amoeba-like cells (Zusso et al., 2012). Amoeba-like microglia have neurotoxic effects that result in decreased phagocytosis and motor ability and the production of inflammatory cytokines (such as reactive oxygen species; Block et al., 2007; Hellwig et al., 2013). At this point, the CNS becomes fertile ground for acute or chronic pathological processes. In recent years, genome-wide association studies (GWAS) have identified a number of genes associated with an increased risk of late-onset AD (LOAD), which are mainly expressed in the innate immune system and microglia (Lambert et al., 2009; McQuade and Blurton-Jones, 2019). Minett et al. (2016) found that in people with dementia, increased microglial activation levels are related to the pathology of AD. By studying human postmortem cortex tissue, Felsky et al. (2019) found that the proportion of morphologically activated microglia is closely related to β-amyloid and tau-related neuropathology and the rate of cognitive decline. In LOAD, the complete loss of functional microglia eventually leads to widespread NFD, dementia and brain failure (Streit et al., 2020). Although the exact mechanism of AD pathogenesis is still unclear, integrated analysis of AD-related genes shows that the immune/microglial gene network has the strongest correlation with AD neuropathology (Zhang et al., 2013). In view of the strong genetic evidence that microglia are involved in AD and that APOE, sex and aging are the main risk factors for AD, we will focus on summarizing their relationships with microglia below.

### APOE and Microglia

#### The Role of APOE in Microglia

APOE is the main cholesterol carrier in the brain, and APOE plays an essential role in lipid transport, cholesterol homeostasis and synaptic stability. In the brain, APOE is mainly secreted by astrocytes, and microglia also produce APOE (Xu et al., 2006). Elevated cholesterol was observed in glial cells lacking APOE, reflecting impaired cholesterol transport in the brain (Nugent et al., 2020). A single-cell RNA sequencing (scRNA-seq) study showed that microglia in the brains of AD mice also exhibited high expression of APOE in the later stage of the disease (Keren-Shaul et al., 2017; Hammond et al., 2019). Interestingly, Krasemann et al. (2017) found through Ingenuity Pathway Analysis (IPA) that APOE is the upstream regulator of MGnD microglia. Treatment of adult microglia with APOE inhibited the specific microglial characteristics of M0 and induce an M1-like phenotype (Butovsky et al., 2015). Notably, when microglia gather around amyloid plaques and release disease-related signals, the expression of APOE in microglia increases significantly (Keren-Shaul et al., 2017; Krasemann et al., 2017).

In humans, there are three main subtypes of APOE (APOE2, APOE3, and APOE4), and they differ only at two amino acid positions (112 and 158; Zhong and Weisgraber, 2009). APOE4 is the largest risk factor for AD, while the relatively rare APOE2 provides AD protection (Corder et al., 1993; Strittmatter et al., 1993). In the process of activating neuronal signals, the three variants of APOE showed differential potentials (APOE4 > APOE3 > APOE2; Huang et al., 2019), reflecting their relative effects on AD risk. The inheritance of different APOE alleles can significantly affect the neurotoxicity caused by the natural immune response of glial cells (Maezawa et al., 2006). As the largest genetic risk factor for sporadic AD, the expression of APOE4 changes the normal function of glial cells, which may increase the risk of AD (Fernandez et al., 2019). Studies of human postmortem brain tissue have found an increase in the number of microglia in AD patients who carry APOE4 (Egensperger et al., 1998; Overmyer et al., 1999). Some studies have pointed out that APOE4 has an inherent impact on microglial physiology by increasing microglial movement and phagocytosis *in vitro*, so it may be the specific cause of microglial dysfunction associated with AD (Muth et al., 2019). *In vitro*, lipopolysaccharide-induced inflammation was the greatest in the presence of APOE4 (Maezawa et al., 2006), and APOE4 caused cell morphology changes (contraction of amoeba morphology and branching process) and the production of pro-inflammatory cytokines (IL-1b, IL-6 and TNF-a, etc.; Vitek et al., 2009; Zhu et al., 2012). It can be seen from the above findings that only carrying the APOE4 gene variant is enough to transform resting microglia into immunologically active microglia (Lin et al., 2018). *In vivo*, APOE4 also increases susceptibility to central and peripheral inflammation in a gene dose-dependent manner (Vitek et al., 2009). Therefore, APOE may be a key factor in microglial disorders in neurodegenerative diseases and may affect AD mainly by regulating microglial activation.

#### The Influence of APOE Subtype on Microglia and Aβ Pathology

**Figure 3:**
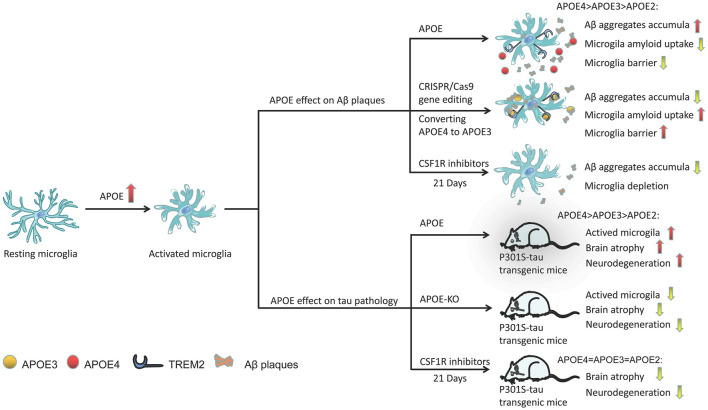
Activation of the role of APOE in microglia under different pathological conditions. APOE in plaques may increase Aβ clearance by binding to TREM2 and activating microglia, promoting microglial migration and phagocytosis, and its efficacy varies with APOE subtype (APOE2 > APOE3 > APOE4). Through CRISPR/Cas9 gene editing, converting APOE4 to APOE3 can enhance the ability of microglia to absorb extracellular Aβ. Similarly, the presence of APOE (especially APOE4) significantly aggravates the neurodegeneration of p301 mice. However, APOE-KO has a powerful neuroprotective effect in tauopathy transgenic mice, reducing the activation of microglia and improving brain atrophy in mice. CSF1R inhibitor-mediated depletion of microglia in P301S-tau transgenic mice can also completely prevent the progression of tau pathology.

Microglia are closely related to Aβ plaques in the AD brain. In AD transgenic mice, there is a large amount of microglial infiltration in Aβ plaques, which are usually the focus of microglial aggregation and activation (Simard et al., 2006; Prokop et al., 2013). Activated microglia mainly exist in and around neurons, or core plaques (Rozemuller et al., 1989; Mackenzie et al., 1995), but not in diffuse amyloid deposits (Perlmutter et al., 1992; Giulian et al., 1995). In fact, diffuse amyloid plaques usually appear in the brains of older people with normal cognitive function, while dense core plaques, especially those associated with dystrophy, are most common in the brains of patients with AD dementia (Serrano-Pozo et al., 2011). In fact, microglial APOE may be the main source of APOE associated with amyloid plaques (Parhizkar et al., 2019). Microglia play a role in the production of Aβ aggregates, and APOE4 may enhance the ability of microglia to participate in this pathological process (Najm et al., 2020). In a study, in which 5XFAD mice were bred with APOE3- or APOE4-targeted replacement mice, the mice expressing APOE4 showed significantly larger and more abundant amyloid plaques and more microglial dystrophy (Figure 3; Yang et al., 2013). APOE4-expressing microglia in the brains of APOE4 knock-in (APOE4-KI) mice may impair the phagocytosis of Aβ aggregates, leading to increased accumulation of Aβ aggregates (Figure 3; Lin et al., 2018; Najm et al., 2020).

Microglia in mouse and human brains can accumulate aggregated Aβ intracellularly, which may be the critical first step in plaque formation (Spangenberg et al., 2019). Early and long-term (21 days) use of colony-stimulating factor 1 receptor (CSF1R) inhibitors (PLX3397) significantly reduced the activation of microglia and inhibited the accumulation of amyloid in nerves, and neuritis plaques (Figure 3; Sosna et al., 2018). However, interestingly, some studies have shown that microglia play a beneficial role in densifying Aβ plaques and protecting neurites from damage (Condello et al., 2015; Wang et al., 2016; Yuan et al., 2016). Microglia can form a protective barrier around amyloid deposits to isolate plaques from surrounding axons, thus preventing plaques from expanding outward and exerting toxic effects on neighboring neurons (Condello et al., 2015; Shi and Holtzman, 2018). The application of high-resolution confocal imaging and *in vivo* two-photon imaging in AD mice indicated the existence of this protective barrier in microglia and showed that it led to the formation of dense plaques with low Aβ affinity (Condello et al., 2015). As a new high affinity ligand of TREM2, APOE may direct microglia to target plaques by interacting with triggering receptor expressed on myeloid cells 2 (TREM2; Figure 3; Cudaback et al., 2011; Shi and Holtzman, 2018; Parhizkar et al., 2019). Yuan et al. (2016) proposed that the TREM2-expressing microglial barrier restricts the growth of amyloid plaques and protects neurons from damage. In the absence of TREM2, Aβ plaques were more diffuse and had lower density, resulting in greater neurite damage (Wang et al., 2016), but had no significant effect on the steady-state plaque load (Fitz et al., 2020). Therefore, TREM2 deficiency prevents the formation of protective barriers of microglia that regulate the compaction of amyloid plaques (Yuan et al., 2016), resulting in the accumulation of dystrophic neurites near diffuse plaques (Wang et al., 2015; Jay et al., 2017; Ulland and Colonna, 2018; Parhizkar et al., 2019). In the presence of APOE4, the coverage of Aβ plaques by microglia was also reduced, resulting in a significantly higher percentage of Aβ in the hippocampus and cortex (Yang et al., 2013). Therefore, we speculate that the presence of APOE4 abolishes the beneficial effect of TREM2 on the microglia barrier. A recent study found that in human samples, either the pathological effect of AD was stronger than that of the APOE genotype or the effect of APOE4 was dependent on AD (Zhao et al., 2020). Therefore, without an increase in familial AD (FAD) mutations, APOE3-KI and APOE4-KI mice did not produce Aβ aggregates in the brain (Zhao et al., 2020). However, transforming APOE4 into APOE3 by CRISPR/Cas9 gene editing enhanced the ability of microglia to take up extracellular Aβ (Figure 3; Lin et al., 2018).

#### The Influence of APOE Subtype on Microglia and Tau Pathology

Friedberg et al. (2020) found that the density of microglia was significantly correlated with tau pathology, and the existence of tau aggregates in reactive microglia was confirmed in the brains of patients (Odawara et al., 1995; Hopp et al., 2018). In fact, PET images in mice showed that tau accumulated before the activation of microglia, and severe brain atrophy occurred after microglial activation (Ishikawa et al., 2018). In the tauopathy mouse model, microglia participate in the process of neurodegeneration which are the main driving force of neurodegeneration (Leyns et al., 2017; Shi et al., 2019). Activated microglia not only aggravated the pathological changes of tau but also drove the accumulation of hyperphosphorylated tau (Vogels et al., 2019), which is consistent with the result that activated microglia co-cultured with primary neocortical neurons significantly increased neuronal tau phosphorylation (Li et al., 2003).

In the P301S-tau transgenic dementia mouse model, mice carrying human APOE4 exhibited more significant brain atrophythan mice carrying other APOE variants, such as APOE2 and APOE3 (Shi et al., 2017). Individuals carrying APOE4 have microglial dysfunction. Moreover, compared with mice expressing APOE2 or APOE3, microglia-mediated cell damage was greater in mice expressing APOE4 (Maezawa et al., 2006). The presence of APOE, especially APOE4, significantly aggravated neurodegeneration in P301S mice (Figure 3; Shi et al., 2017). However, APOE knockout (APOE-KO) had a strong neuroprotective effect in tauopathy mice (Figure 3; Shi et al., 2019), significantly reducing the activation of microglia and improving brain atrophy (Figure 3; Shi et al., 2017). Microglia in APOE-KO mice still engulfed dead neurons, but the MGnD phenotype did not appear (Keren-Shaul et al., 2017; Krasemann et al., 2017; Bisht et al., 2018), suggesting that APOE is essential to maintaining this phenotype. The microglia of P301S-tau transgenic mice aged 6–9.5 months were depleted by PLX3397, which completely blocked the progression of tau pathology (Figure 3; Shi et al., 2019). However, using the same method to reduce microglia in the aged mouse model of invasive tau disease did not lead to changes in tau lesions or neurodegeneration (Bennett et al., 2018). In addition, a slight reduction in microglia (~30%) did not change the tau load or cortical atrophy in the mouse model of tau disease (Bennett et al., 2018). Controlling the activity of microglia may not be an effective strategy against the pathological changes of tau because higher doses of PLX3397 or early intervention may be required during the course of the disease.

### The Influence of Sex on Microglia and AD

The sex difference in microglial function may be one of the factors affecting the differences observed between men and women in the susceptibility and prognosis of neurodevelopmental and neuropsychiatric diseases (Figure 1). Compared with males, females have more phagocytic microglia (Figure 1; Nelson et al., 2017). After treatment with estradiol, the hormone responsible for masculinizing the rodent brain, the number of phagocytosing microglia in female mice decreased to the typical level observed in male mice (Figure 1; Nelson et al., 2017). Female microglia expressed lesser proinflammatory genes and had neuroprotective effects in ischemic animal models (Schwarz et al., 2012). However, in APP^NL-G-F^ mice, the microglia of female mice progressed faster on the ARM trajectory than those of male mice (Sala Frigerio et al., 2019). After ovariectomy in APP23 mice (an animal model of chronic neuritis replicated in AD patients), it was found that microglia developed to a high reaction state, and a large number of microglial response markers were upregulated (Vegeto et al., 2006; Benedusi et al., 2012; Sarvari et al., 2012). Increased expression of inflammatory markers was also observed in postmenopausal women, especially in the functional areas of the brain that responded most strongly to inflammation (Lei et al., 2003). This sex-dependent difference in microglial response is interesting because the incidence of AD is higher in women (Laws et al., 2018). Therefore, we speculate that normal female microglia and estrogen have a protective effect, but under the influence of AD, female microglia no longer have a protective effect but instead accelerate the course of female AD.

### The Influence of Aging on Microglia and AD

The average age of onset for sporadic AD is above the age 65 years old, and age is the main risk for AD (Hebert et al., 2003). Microglia deteriorate due to age and cell aging but also due to certain risk factors (as observed in genetically and environmentally induced AD; Figure 2). Resting microglia have neuroprotective effects, but during aging and AD, changes in microglial proliferation, morphology and motility are signs of decreased microglial function (Spittau, 2017). Compared with the microglia of aged AD mice, the microglia of young AD mice significantly lengthened and contracted their protrusions, and their protrusions moved more (Figure 2; Koenigsknecht-Talboo et al., 2008). Microglia in AD usually appear in clusters and gather around fibrous Aβ deposits (Figure 2; Perlmutter et al., 1992; Hickman et al., 2008). With aging, microglia exhibit significantly less process movement, and the coverage of microglia decreases, leading to enlarged protofibrillar Aβ42 hotspots and more serious neuritic dystrophy (Condello et al., 2015). Srinivasan et al. (2020) found that human AD microglia not only upregulated APOE but also showed enhanced characteristics of human aging (Figure 2). Aging and systemic diseases push individual microglia toward the proinflammatory phenotype, which damages the connectivity of the neural network, leading to neuropsychiatric diseases (Valdés-Ferre et al., 2020). Microglia respond to brain tissue damage accumulated during aging and neurodegeneration, resulting in increased inflammatory reactions, disturbances in neuronal communication, and decreases in phagocytosis and motor ability (Figure 2; Hefendehl et al., 2014). In this case, the ability of microglia to monitor the brain and respond to various injuries is weakened (Baron et al., 2014; Udeochu et al., 2016; Pimenova et al., 2017). As expected, neuroinflammation in the brains of aged mice lasted longer than that in the brains of adult mice (Norden et al., 2015). The delayed response of microglia in the brains of aged mice to injury may affect the pathological outcomes of aged animals after brain injury.

By imaging acute brain slices by two-photon microscopy *in vivo*, Krabbe et al. (2013) found that the directed movement and phagocytic activity of microglia in AD mice were also seriously impaired (Figure 2). Compared with the microglia of young mice, the microglia of old mice had larger cell bodies, shorter dendrites, thicker dendrites, fewer branches, and signs of malnutrition (Figure 2; Vaughan and Peters, 1974; Streit et al., 2004; Sierra et al., 2007; Baron et al., 2014). As AD is a disease closely related to aging, it may not be entirely surprising that microglia of the AD brain share some phenotypes with aging microglia. The consequence of these aging- and AD-related proinflammatory phenotypic changes in microglial communication is the impairment of the synaptic plasticity and cognitive ability of neurons (Udeochu et al., 2016). *In vitro* experiments showed that aged microglia could not engulf Aβ as effectively as young microglia (Njie et al., 2012). Compared with those in wild-type control mice, the morphological changes of microglia in aged AD mice were most obvious in the pathological area containing Aβ plaques (Koenigsknecht-Talboo et al., 2008). The above results show that, as a disease closely related to aging, AD causes microglial changes similar to those caused by aging. Aging may increase the sensitivity of AD to microglia, leading to further aggravation of microglial dysfunction in elderly AD.

## The Crosstalk Among APOE, Sex and Aging in Microglia

Notably, in the development of Alzheimer’s disease in reality, the three major risk factors for AD-APOE genotype, sex, and aging do not affect microglia independently. Aging can be clearly defined the greatest risk factors for AD and its effect on microglia are consistent. With aging, APOE genotype and gender contributed to the pathogenesis and development of AD by affecting microglia through synergistic effect.

APOE4 genotype is the strongest genetic factor of sporadic AD. The age of AD onset in APOE4 genotype carriers was earlier than that in non-APOE4 carriers, suggested that the effect of APOE4 genotype on AD onset goes hand in hand with age. APOE4 disrupts normal glial cell biology and intersects with changes that occur during normal aging, resulting in neurodegeneration and cognitive dysfunction (Costantini et al., 2018; Fernandez et al., 2019). A great deal of evidence showed that there is a strong connection between microglial activation in AD patients and the APOE genotype, especially related to aging (Saitoh et al., 1997; Egensperger et al., 1998). Compared with non-aged mice, the microglial proteome of aged mice showed enrichment of the APOE protein (Rangaraju et al., 2018). A proteomic analysis of the postmortem human brain also showed that APOE expression was higher in aged microglia (Olah et al., 2018). In AD and aging mouse models, microglial APOE mRNA was upregulated (Figure 2; Hickman et al., 2013; Orre et al., 2014). These results suggested that aging alone is sufficient to induce APOE expression in microglia at the mRNA and protein levels, then accelerating the impact of APOE on the development of AD.

Interestingly, there is a gender effect that women with APOE4 carriers at higher risk for AD-related pathology, amyloid plaques, and neurofibrillary tangles (Corder et al., 2004). ARM cells are the dominant microglial cell type in APP^NL-G-F^ mice, while the proportion of ARM cells in females is higher than that in males, and the response is earlier and more pronounced, especially in older mice (Sala Frigerio et al., 2019), suggested that the ARM response may be the intersection of APOE4 genotype, sex, and aging in AD. It is noted that plaque compaction is a beneficial result of the interaction between microglia and plaques, but the APOE4 genotype and female were showed associated with lower plaque compaction (Stephen et al., 2019). Microglial coverage of plaques was highest in male APOE3 mice, while the microglial coverage observed in APOE4 mice and female mice was significantly lower, meanwhile the amyloid level was increased (Stephen et al., 2019). It has been reported that the aged microglia of female mice may lost the ability to adapt their phagocytosis to inflammatory conditions (Yanguas-Casas et al., 2020). With aging, estrogen resistance may be related to the impaired ability of microglia to reduce inflammation. Although carrying the APOE4 genotype is not necessary or sufficient for the development of AD, these observations may indicate that ARM increases the vulnerability of this critical pathway, especially in older women with APOE4 carriers. Therefore, we speculate that ARM cells may further aggravate AD pathology by synergistic interaction with APOE4 genotype, low circulating estrogen in aging women, and pro-inflammatory factors induced by aging, AD pathology may worsen with the increase of ARM cells in female APOE4 carriers.

In summary, aging is the driving factor that expanding the APOE4 or gender factor for AD risk, and it is no doubt that microglia play the central role to affect the occurrence and development of AD between the interactions of these risk factors. In future, a more comprehensive understanding of the mechanisms that contribute to the increased risk of AD is critical to developing interventions and determining who will benefit the most.

## Conclusions and Perspectives

Microglia are essential for maintaining normal brain function and have been recognized as having a significant role in neurodegenerative diseases, such as AD. As microglia colonize the brain early in embryonic development, environmental and/or genetic disturbances may alter the development, synaptic pruning and surveillance of microglia or cause other pathologies that may directly or indirectly lead to neurodevelopmental and neuropsychiatric diseases. In general, the role of microglia in alleviating or promoting the pathological development of AD may be mediated by the specific factors, they release and the balance between proinflammatory and anti-inflammatory cytokines. As the resident immune cells in the CNS, microglia play a key role in maintaining brain homeostasis. Genetic studies have shown that microglia play an essential role in the pathogenesis of AD. In general, microglia can remove harmful cell debris. When Aβ levels accumulate, microglia phagocytose and clear Aβ aggregates. When the capacity of this activity is exceeded, microglia form a barrier that tightly envelops Aβ aggregates in dense core plaques and separates them from neurons. These activated microglia are dependent on TREM2 and assisted by APOE. However, in response to the activation of microglia, the two hallmark AD pathologies, Aβ plaque and tau pathology, may show opposite trends. Sometimes, due to aging or genetic susceptibility, the function of these microglia is insufficient to prevent the occurrence and development of AD.

The aging of the global population has led to a rapid increase in the incidence of AD, and there is a growing need for an effective therapy for AD to improve the quality of life of elderly patients with AD. Microglia are considered to be the key mediators of neuroinflammation during AD. Changing the function of microglia in disease states is the subject of in-depth research and provides opportunities for developing innovative AD treatments. These include therapeutic strategies to inhibit the inflammatory response of microglia (e.g., with NLRP3 inhibitors and RIPK1 inhibitors; Heneka et al., 2018; Yuan et al., 2019), enhance microglial function (e.g., with antibodies to the extracellular region of TREM2: AL002a and 4D9; Cignarella et al., 2020; Schlepckow et al., 2020), enhance lysosomal function in microglia (Majumdar et al., 2011) and promote lipid processing in microglia (e.g., with agonists of nuclear receptors; Moutinho and Landreth, 2017) and clearance of diseased microglia (Han et al., 2017). What needs special attention is that recently many pharmacological strategies have been developed to successfully eliminate microglia from the central nervous system (Table 1) such as the use of diphtheria toxin (Bruttger et al., 2015), clodronate liposomes (Faustino et al., 2011; Han et al., 2019), and CSF1R inhibitors (mainly PLX3397 and PLX5562; Elmore et al., 2014). Although in a variety of AD in mice, decrease of microglia CSF1R antagonists have different results, but most studies have found that microglia reduce improved cognition (Dagher et al., 2015; Spangenberg et al., 2019), saved dendritic spine loss (Dagher et al., 2015; Spangenberg et al., 2019), and reduced overall neuroinflammation and neuropathic plaque formation (Sosna et al., 2018), even blocking the Tau pathology process (Table 1; Shi et al., 2019). CSF1R inhibitors are the effective tools to achieve microglial depletion. Microglia account for 30%–99% of all cells could be eliminated by preparing different concentrations of inhibitors in food, so as to achieve comprehensive control of microglia population. Moreover, removal of the CSF1R inhibitor from mice with depleted microglia caused the entire CNS to rapidly reproduce new cells within 14 days (Elmore et al., 2014), and the new renewal of microglia ultimately has a good benefit on the treatment of AD.

**Table 1:** Summary of pharmacological microglia depletion approaches.

Pharmacological intervention	Animal model	Efficiency	Time window	Physiological effects	References
CSF1R inhibitor (PLX3397; 290 mg/kg)	Wild-type mice (2/12/18-month-old)	~99%	21 days	No cognitive/behavioral impairments. Inhibitor cessation (14 days) induced cell repopulation.	Elmore et al. (2014); Elmore et al. (2015)
CSF1R inhibitor (PLX3397; 290 mg/kg)	Tauopathy mice (12-month-old).	~30%	21 days	No change in tau burden, cortical atrophy, blood vessels.	Bennett et al. (2018)
CSF1R inhibitor (PLX3397; 400 mg/kg)	P301S tau transgenic mice (6-month-old)	90%–100%	7–21 days	Blocked the progression of pathological tau stages.	Shi et al. (2019)
CSF1R inhibitor (PLX3397; 290 mg/kg)	5xfAD mice (10-month-old)	~99%	21/28 days	Reduced intraneuronal amyloid. Improved cognitive function. Prevent neuronal loss and contextual memory.	Spangenberg et al. (2016) and Sosna et al. (2018)
CSF1R inhibitor (PLX5622; 1,200 mg/kg)	5xfAD mice (10-month-old)	~80%	28 days	Reduced dendritic spine loss, prevent neuronal loss and contextual memory.	Spangenberg et al. (2016)
CSF1R inhibitor (PLX5622; 300/ 1,200 mg/kg)	3xfAD mice (15-month-old)	30%–95%.	7/21 days	Lower dose inhibition prevented microglial association with plaques. Lower dose inhibition improved cognition.	Elmore et al. (2014)
Clodronate Liposomes (7 μg/μl injection	Cx3cr1^GFP/ + ^ mice (8–10-week-old)	35%–85%	1~ 3 days	Damaged other brain cells. Damaged the integrity and density of blood vessels.	Han et al. (2019)
Diphtheria toxin (DT; 500 ng DT injection)	Cx3cr1^CreER^ mice (8-week-old)	~80%	<5 days	Induced cytokine storm. Reproduced within 5 days after microglia failure.	Bruttger et al. (2015)

This treatment strategy also has limitations and concerns. Since microglia are the main immune cells of the CNS, long-term elimination of human microglia is currently not feasible (Butowski et al., 2016). In view of the effect of CSF1R inhibitors, the ability to replace microglia by stopping CSF1R inhibitor treatment also has obvious clinical value, whether it is possible to delay the course of AD by eliminating the microglia after extensive nerve damage and allowing them to completely refill the CNS depends on whether new cells can replace the microglia lost in brain. The current clinical use of CSF1R inhibitors further confirms that short-term administration of CSF1R inhibitors can effectively eliminate human microglia (Butowski et al., 2016). Therefore, short-term elimination of microglia and cell proliferation may be a clinically feasible and novel method to solve neuroinflammatory events and promote brain recovery in brains affected by microglial dysfunction (Rice et al., 2017; Son et al., 2020). However, Since the recently obtained information on the different roles of microglia mainly comes from studies in mouse disease models, it is necessary for us to develop new disease models, including human induced pluripotent stem cells (iPSCs; Muffat et al., 2016; Bohlen et al., 2017; Gosselin et al., 2017), to fully understand which findings can be translated from mice to humans.

Here, the increased understanding of APOE genotypes, sex and aging in microglia provides a new, microglia-focused therapeutic approach for AD that is fundamentally different from current methods for Aβ or tau. The discovery of sex differences in susceptibility provides a basis for sex-related therapy, which must be based on a better understanding of the relationships among the endocrine, immune, and nervous systems of young and aged individuals. Microglia replacement may be a new method applicable in many cases, especially in the elderly AD population. Microglia extensively manipulate the structure and function of neurons to produce beneficial results. These results provide new insights into the behavior of microglia. Since intervention in the late stages of dementia is unlikely to change the disease, treatment should take into consideration the disease stage, and be designed to alleviate neuroinflammation according to the exact disease stage, which would provide the possibility for the development of effective treatments targeting microglia. Whether microglia depletion and subsequent regeneration is a promising therapeutic strategy for AD, is still an open question. More research is needed to clarify the extent to which aged microglia can be restored to their protective functions and reset their aging clock. The beneficial and harmful effects of the microglia rejuvenation strategy still need to be considered to promote the development of safe and effective microglia-targeted therapies. In the future, replacing or manipulating microglia, combined with the transplantation of iPSC-derived neurons, will give us the opportunity to analyze these two possibilities.

## Author Contributions

YC wrote the review and designed the figures. YW and TH critically revised the manuscript. YW and FC instructed the manuscript and designed the figures. YS revised the manuscript. LC formulated the original idea, revised and approved the manuscript. All authors contributed to the article and approved the submitted version.

## Conflict of Interest

The authors declare that the research was conducted in the absence of any commercial or financial relationships that could be construed as a potential conflict of interest.

## Abbreviations

AD: Alzheimer’s disease
Aβ: amyloid β
CNS: central nervous system
APOE: apolipoprotein E
iPSC: human pluripotent stem cell
DAM: disease-associated microglia
MGnD: neurodegenerative microglia
ARM: activation response microglia
GWAS: genome-Wide association studies
LOAD: late-onset AD
IPA: Ingenuity pathway analysis
APOE4-KI: APOE gene knock-in
TREM2: triggering receptor expressed on myeloid cells 2
FAD: familial AD
APOE-KO: APOE gene knockout
CSF1R: Fractalkine Receptor
NLRP3: nod-like receptor protein 3 inflammasome
RIPK1: Receptor Interacting Protein Kinase 1
IRF8: interferon regulatory factor 8
A2M: alpha-2-macroglobulin antisense RNA 1
CHI3L1: chitinase 3-like 1.
